# Combining whole-genome shotgun sequencing and rRNA gene amplicon analyses to improve detection of microbe–microbe interaction networks in plant leaves

**DOI:** 10.1038/s41396-020-0665-8

**Published:** 2020-05-13

**Authors:** Julian Regalado, Derek S. Lundberg, Oliver Deusch, Sonja Kersten, Talia Karasov, Karin Poersch, Gautam Shirsekar, Detlef Weigel

**Affiliations:** 1 Department of Molecular Biology, Max Planck Institute for Developmental Biology, 72076 Tübingen, Germany; 2 Institute of Plant Breeding, Seed Science and Population Genetics, University of Hohenheim, 70599 Stuttgart, Germany

**Keywords:** Metagenomics, Microbiome

## Abstract

Microorganisms from all domains of life establish associations with plants. Although some harm the plant, others antagonize pathogens or prime the plant immune system, support the acquisition of nutrients, tune plant hormone levels, or perform additional services. Most culture-independent plant microbiome research has focused on amplicon sequencing of the 16S rRNA gene and/or the internal transcribed spacer (ITS) of rRNA genomic loci, which show the relative abundance of the microbes to each other. Here, we describe shotgun sequencing of 275 wild *Arabidopsis thaliana* leaf microbiomes from southwest Germany, with additional bacterial 16S and eukaryotic ITS1 rRNA amplicon data from 176 of these samples. Shotgun data, which unlike the amplicon data capture the ratio of microbe to plant DNA, enable scaling of microbial read abundances to reflect the microbial load on the host. In a more cost-effective hybrid strategy, we show they also allow a similar scaling of amplicon data to overcome compositionality problems. Our wild plants were dominated by bacterial sequences, with eukaryotes contributing only a minority of reads. Microbial membership showed weak associations with both site of origin and plant genotype, both of which were highly confounded in this dataset. There was large variation among microbiomes, with one extreme comprising samples of low complexity and a high load of microorganisms typical of infected plants, and the other extreme being samples of high complexity and a low microbial load. Critically, considering absolute microbial load led to fundamentally different conclusions about microbiome assembly and the interaction networks among major taxa.

## Introduction

Microorganisms affect many important plant traits. Opportunistic microbes can slow down growth or kill a plant, while beneficial ones can prime the plant immune system [1], directly antagonize pathogens [2], or indirectly inhibit pathogens by contributing to a suppressive environment [3]. Microbes may adjust plant hormone levels [4] and participate in nutrient acquisition [5, 6], among other processes [7]. Research in this area has revealed that most of the organisms present on and in healthy plant leaves are typically bacteria, and often Proteobacteria such as *Sphingomonas*, *Pseudomonas*, and *Methylobacterium* [8–10]. With the exception of specific pathogenic strains, other microbes such as archaea, fungi, and protists including oomycetes generally have lower abundance on leaves than bacteria. They have also received less attention because they are less easily cultured and are genetically more complex. Due to the difficulty of adequately capturing microbial complexity and diversity, we still lack a good understanding of the composition and dynamics of leaf microbial communities, their absolute abundances on the plant, and how they relate to other aspects of host biology such as genotype, location, or environmental conditions.

Amplicon sequencing, in which a specific locus common to a target group of organisms, usually the 16S rRNA gene (or hereafter rDNA) for bacteria and ITS for fungi, is amplified and sequenced, has been the tool of choice for revealing the taxonomic composition of a microbiome. Albeit usually very informative, amplicon sequencing relies on oligonucleotide primers that can disfavor or exclude some organisms, with the consequence that some taxa are systematically ignored. Perhaps even more importantly, there is also no information on the absolute abundances of taxa [11–13]. Furthermore, one gene cannot reliably predict the other genes and genetic and metabolic functions in a microbe beyond some conserved features [14, 15]. Whole-metagenome shotgun sequencing of DNA extracts has become an attractive alternative for dissecting complex microbial communities, and databases and algorithms that support such efforts are being developed [16, 17]. Shotgun sequencing supplies information on the total DNA content of microorganisms as opposed to a specific locus, which in principle enables the functional characterization of reads, de novo assembly of genes, and in the best cases, leads to the recovery of entire metagenome assembled genomes [8, 18–20].

We used metagenome sequencing to characterize the leaf-associated (phyllosphere) microbiome of 275 wild *Arabidopsis thaliana* individuals from around Tübingen in southwest Germany, at four different timepoints between 2014 and 2016. Of these, we subjected 176 to 16S rDNA and ITS1 sequencing. We achieved low (<100 Mb) to high (>1 Gb) depths of microbe-associated metagenomic sequences per sample. Because de novo assembly turned out not to be a fruitful avenue for these data, we relied on mapping reads to reference databases. We document the relative abundance of eukaryotic, archaeal, and bacterial microbes, revealing a small but noteworthy presence of fungi and oomycetes. Unsurprisingly, the wild *A. thaliana* microbiota was highly variable between individuals, but there were some clear patterns in the dominant microbes; in particular *Pseudomonas* dominated in some sites and *Sphingomonas* in others. We estimated absolute microbial load as the ratio of microbial reads to plant chromosomal reads in each sample, and observed that the microbial load varied across samples from <1% to up to 77% of plant reads. We used the bacterial load calculated from the metagenome data to similarly scale the 16S data. After producing load-corrected tables, we observed that intertaxa abundance correlations often changed in sign compared with compositional amplicon data and metagenome data, in which microbial reads had been normalized by total sum scaling. Similar observations were made in human gut microbiomes after microbial load information was incorporated [21]. These results underscore the importance of measuring microbial load, either using deep shotgun sequencing, or, as we propose, combining low coverage shotgun data with amplicon data in a cost-effective hybrid approach.

## Methods

### Sampling, processing of plants, and metagenomic library preparation

Plants were sampled from previously described sites Gniebel, Eyach (EY), Pfrondorf (PFN), and Jugendhaus Einsiedel (JUG) around Tübingen, Germany [22, 23], in four distinct sampling batches, which also had different processing details, representing our evolving pipeline.

#### Batch 0—single plant

A plant visibly infected with both *Hyaloperonospora arabidopsidis* and *Albugo* sp. was collected in Fall 2014 from the village of Gniebel (48° 34′ 34.10″ North Lat., 9° 10′ 55.42″ East Long.) using sterile tweezers and scissors, placed in a sterile 15 mL tube, and brought back to the lab on ice where it was frozen at −80 °C until further processing. The frozen plant was ground in the presence of liquid nitrogen using a mortar and pestle that was lined with four layers of autoclaved aluminum foil. Approximately 250 g of the resulting powder was used for DNA extraction, using a custom protocol we previously described [22]. Briefly, the sample was subjected to bead-beating in the presence of 1.5% sodium dodecyl sulfate (SDS) and 1 mm garnet rocks, followed by SDS cleanup with 1/3 volume 5 M potassium acetate, and then SPRI beads. The library was prepared using the TruSeq Nano kit (Illumina, San Diego, CA, USA), with DNA shearing performed with a S2 focused ultrasonicator (Covaris, Woburn, MA, USA) as suggested in the manufacturer’s protocol. Rather than Illumina adapters, we used custom adapters described in ref. [24]. The sample was sequenced on one lane of a HiSeq 2000 instrument (Illumina), using a 100 bp single-end kit.

#### Batch 1—nine plant test of shearing methods

Nine plants were sampled from EY in late December 2014. Rosettes were collected in 50 mL tubes with flame-sterilized scissors and tweezers and brought back to the lab for processing. In the lab, three rosettes were left unwashed, three were washed in sterile water, and three were washed in Silwet L-77 solution. Rosettes were then snap frozen and ground to a fine sand-like consistency with sterile aluminum foil-lined mortar and pestles, as described above. For large rosettes, the ground plant material was transferred among up to three DNA-extraction tubes which were processed in parallel to better represent the sample, and pooled again prior to library preparation. The DNA was extracted as described above for the batch 0 plants. Two sets of libraries were made for the nine plants using homebuilt protocols: one sheared with a Covaris instrument, and one sheared with Shearase enzyme.

##### Covaris based

For one set of libraries, 100 ng of DNA in 130 μL of elution buffer was sheared on a S2 focused ultrasonicator (Covaris) for 65 s using intensity = 4, duty cycle = 10%, and 200 cycles per burst, to yield a fragment size of ~350 bp. The sheared DNA was cleaned with SPRI beads in a 0.8:1 bead to sample ratio, and eluted in 15 μL. End repair, A-tailing, and adapter ligation were performed similar to ref. [25] following “Alternative Protocol 2” with double DNA size selection after the end repair step, and using homemade SPRI beads instead of AMPure XP beads. Other minor modifications were that the total volume of the end repair reaction was scaled down to ¼ volume, with DNA eluted after SPRI-cleanup in 17 μL. The total volume of the A-tailing reaction was scaled down to ½ volume. Again, custom adapters described in ref. [24] were substituted for Illumina adapters.

##### Shearase based

For the second set of libraries, 100 ng of DNA in 20 μL of EB buffer was mixed with 9.5 μL of 3X reaction buffer and 0.5 μL of dsDNA Shearase Plus (Zymo Research, Freiburg, Germany), and incubated for 30 min at 37 °C to yield a size range between 200 and 1000 bp, before the reaction was stopped by addition of 3 μL EDTA. The shared DNA was cleaned with SPRI beads and eluted in 17 μL EB. Size selection, A-tailing, and adapter ligation were performed exactly as with the Covaris-based protocol. Final cleaned libraries prepared using both Covaris and Shearase protocols were quantified with PicoGreen (Invitrogen, Carlsbad, CA, USA) using 1 μL of DNA in 100 μL reactions, and molecules were pooled in equimolar amounts. The pooled library was size selected for 350–700 bp final insert size on a Blue Pippin instrument (Sage Science, Beverly, MA, USA). All samples were sequenced on an Illumina HiSeq 3000 instrument with 2 × 150 paired-end reads.

#### Batch 2—set of 90 plants

Plants were collected from EY and PFN in Fall 2014 (November 24 and 25) and Spring 2015 (March 18 and 19). All samples were brought back to the lab in 50 mL tubes, washed 3× in sterile water to remove adhering dust and soil, and then flash-frozen and stored at −80 °C until they were ground in sterile foil-covered mortar and pestle. DNA was extracted as described for other plants above. Metagenomic libraries were prepared as described for Covaris-sheared libraries from batch 1; the entire set of 90 libraries was quantified, combined to one pool, size selected, and sequenced on an HiSeq 3000 instrument with 2 × 150 paired-end reads over multiple lanes.

#### Batch 3—set of 176 plants

Plants were harvested from EY (11 Dec. 2015 and 23 Mar. 2016), JUG (15 Dec. 2016 and 31 Mar. 2016), and PFN (31 Mar. 2016). Whole rosettes were removed with sterile scissors and tweezers, and washed 3× with sterile water. Two leaves were removed and independently processed to culture bacteria as previously published in [22], and the remaining rosette was flash-frozen on dry ice and processed for metagenomic sequencing and 16S rDNA sequencing of the V4 region. The metagenomic libraries were prepared using a modification of the Nextera protocol for smaller volumes similar to ref. [26], as previously described [22]. As for batch 1 and batch 2 plants, the full set of 176 libraries was quantified and combined to one pool, size selected for 350–700 bp final insert size, and sequenced on an HiSeq 3000 instrument with 2 × 150 paired-end reads over multiple lanes.

### 16S rDNA V4 library construction and sequencing for batch 3 plants

Amplicon sequencing of the V4 region of the 16S rDNA gene performed using a two step PCR protocol using PNAs to block chloroplast and mitochondrial sequences, slightly modified from ref. [27]. The first PCR step amplified the rDNA using 515F [28] and 806R [29] primers as well as short overhangs (Supplementary Table 1) and a second step primed these overhangs to added Illumina adapters. The primers differed from Lundberg et al. [27] in two key ways. First, although the frameshifting nucleotides were kept, the molecular tagging nucleotides were removed from the primers to make the protocol simpler and robust to more variable DNA quantities. Second, the primers were modified such that the Illumina TruSeq priming sequences were used on both the forward and reverse primers, as opposed to the use of a Nextera sequence on the forward primer. Unique barcoding of samples was accomplished by use of 96 independent indexing primers in the second PCR, combined with two combinations of frameshift primers in the first PCR as explained in ref. [27]. Half of the samples from the first PCR were amplified with 515F frameshifts 1, 3, and 5 paired with 806R frameshifts 2, 4, and 6. The other half of the samples from the first PCR paired 515F frameshits 2, 4, and 6 with 806R reverse frameshifts 1, 3, and 5. This strategy allowed up to 192 samples to be uniquely indexed.

In the first PCR, each reaction was prepared in 60 μL, which was split into three 20 μL reactions run in parallel for 29 cycles. Three parallel reactions helps mute the influence of stochastic bias that might affect any single reaction. The 60 μL mix contained 6 μL of TAQ buffer (NEB, Ipswich, MA, USA), 3 μL of 5 μM forward primers mix, 3 μL of 5 μM reverse primers mix, 0.45 μL of 100 μM pPNA, 0.45 μL of 100 μM mPNA, 1.2 μL of 10 mM dNTPs), 0.48 μL of Taq polymerase (NEB), 40.4 μL of PCR-grade water, and 50 μL of template DNA. The first PCR was run for 94 °C for 2 min followed by 29 cycles of 94 °C for 30 s, 78 °C for 5 s, 50 °C for 30 s, and 72 °C for 1 min, and finally 72 °C for 2 min. The three 20 μL reactions were pooled and 5 μL was run on a gel to confirm amplification. The remaining 55 μL were cleaned with 55 μL of SPRI beads [30] at a bead:sample ratio of 1:1 to remove PCR primers, the DNA was resuspended in 30 μL of water. Between 1 and 5 μL of this product from the first PCR, based on gel band intensity, was used in the second PCR of six cycles to add illumina adapters.

The second PCR was prepared in 25 μL, and contained 5 μL of Q5 PCR buffer (NEB), 0.0625 μL of 100 μM universal forward primer, 1.25 μL of 5 μM barcoded reverse primer, 0.5 μL of 10 mM dNTPs, 0.25 μL of Q5 polymerase (NEB), 12.875 μL of PCR-grade water, and 5 μL of water + DNA from the first PCR. The second PCR was run for 94 °C for 1 min followed by six cycles of 94 °C for 20 s, 60 °C for 30 s, and 72 °C for 30 s, and finally 72 °C for 2 min. Successful addition of adapters was confirmed by 5 μL of the final product from each reaction on an agarose gel, allowing visualization of a size shift. Final amplicons averaged 430 bp in length. The remaining 20 μL of product was cleaned with SPRI beads and resuspended in 40 μL of EB. Libraries were quantified by PicoGreen in 100 μL reactions, pooled in equimolar amounts, and sequenced using on a MiSeq instrument with a V2 2 × 250 bp reagent kit (Illumina), which was sufficient to overlap and assemble the forward and reverse reads. The frameshifts built into the primers used in the first PCR made the addition of PhiX to increase sequence diversity unnecessary [27].

### ITS1 library construction and sequencing for batch 3 plants

ITS1 rDNA amplicons were prepared similarly to 16S rDNA amplicons, using gene-specific primers for the first PCR and adding indexes and adapters in the second PCR. We used a protocol modified from ref. [31], which uses blocking primers to prevent amplification of plant sequences. Because blocking primers, unlike PNAs, result in a quantifiable PCR product, we used the cycling conditions suggested in [31] to prevent the product of the blocking product from reaching detectable levels. As with the 16S rDNA protocol, we used six frameshifting forward ITS1F primers and six frameshifting reverse ITS2R primers. The first 60 μL PCR reaction (also run as three parallel 20 μL reactions) included 6 μL of 10X ThermoPol Taq buffer (NEB), 0.96 μL of 5 μM forward primer (0.08 μM final), 0.96 μL of 5 μM reverse primer (0.08 μM final), 0.15 μL of 100 μM forward blocking primer (0.25 μM final), 0.15 μL of 100 μM reverse blocking primer (0.25 μM final), 1.2 μL of 10 mM dNTPs, 0.48 μL of Taq DNA polymerase (NEB), 45.1 μL of PCR-grade water, and 5 μL of DNA. The first PCR ran for 94 °C for 2 min followed by ten cycles of 94 °C for 30 s, 55 °C for 30 s, and 72 °C for 30 s, and finally 72 °C for 3 min. The three 20 μL reactions were pooled, cleaned with a bead:sample ratio of 1:1 to remove PCR primers, and resuspended in 30 μL of water.

The second PCR was prepared in 25 μL, and contained 5 μL of Q5 PCR buffer (NEB), 0.0625 μL of 100 μM universal forward primer, 1.25 μL of 5 μM barcoded reverse primer, 0.5 μL of 10 mM dNTPs, 0.25 μL of Q5 polymerase (NEB), 4.875 μL of PCR-grade water, and 13 μL of DNA from the first PCR. The second PCR was run for 94 °C for 1 min followed by 25 cycles of 94 °C for 20 s, 60 °C for 30 s, and 72 °C for 30 s, and finally 72 °C for 2 min and cool down to room temperature. Successful PCR and addition of adapters was confirmed by 5 μL of the final product from each reaction on an agarose gel, with the major band produced around 400 bp in length. The remaining 20 μL of product was cleaned with SPRI beads and resuspended in 40 μL of elution buffer. Libraries were quantified with PicoGreen in 100 μL reactions, pooled in equimolar amounts, and sequenced using a MiSeq V3 2 × 300 reagent kit (Illumina).

### Amplicon quality processing, clustering, and classification

Raw sequences from both 16S and ITS1 rDNA amplicons were first demultiplexed according to their 9 bp barcodes added in the second PCR, not allowing any mismatches. All sequences were further demultiplexed by the frameshift combinations using strict regular expressions without mismatches in any part of the primer sequence (https://github.com/derekLS1/Metagenome2019). Forward frameshifts 1, 3, and 5 were only allowed pairings with reverse frameshifts 2, 4, or 6. Forward frameshifts 2, 4, and 6 were only allowed pairings with reverse frameshifts 1, 3, and 5.

Forward and reverse reads from the 16S rDNA sequences were merged with FLASH [32] using a minimum overlap set to 30 bp and (-m 30). Most ITS1 amplicons were small enough to overlap with these longer reads, but some reads were longer and overlap was not possible, so only the forward read was used for downstream analyses (read 1), although the frameshift in read 2 was used for demultiplexing.

All primer sequences were removed. Because of the frameshifts in the primer sequences, ITS1 read 1 sequences had variable lengths after removing primers, and therefore all were trimmed to a common length of 271 bases before clustering. Additional quality filtering, removal of chimeric sequences, OTU preparation and OTU tables, and taxonomic assignment were done with USEARCH10 [33] (https://github.com/DerekLS1/Metagenome). OTUs were prepared at 100% as “zero-radius OTUS” (zOTUS, a form of Amplicon Sequence Variant) [34]. The 16S rDNA taxonomy was based on the RDP training set v16 (13k seqs.), and ITS1 taxonomy was based on UNITE USEARCH/UTAX release v7.2 (UNITE Community, 10.15156/BIO/587476).

### Metagenome read QC and host data removal

Sequencing libraries were subject to adapter trimming and quality control with Skewer [35]. Reads were trimmed to a minimum length of 30 bp and minimum average Phred score of 20. After sequencing, samples were composed of a mixture of mostly host *Arabidopsis thaliana* reads and microbial origin reads. In order to remove most of the plant reads, libraries were aligned against the *A. thaliana* reference genome [36] using the bwa-mem algorithm with standard parameters [37]. After mapping, only read pairs for which neither of the mates mapped against the plant reference genome were mapped against the metagenomic reference. Data aligned to the host was later used for host plant genotyping.

### Metagenomic profiling

Using DIAMOND [38] with default mapping parameters, the putative microbial reads were mapped against the entire NCBI nr protein database (March 2018), which includes protein sequences from all three domains of life and viruses. In order to keep analysis time and file sizes manageable, a maximum of 25 matches per sequencing read was permitted. Diamond analysis archive files were then parsed for taxonomic binning with MEGAN [39]. Reads were binned to different taxa using the weighted lowest common ancestor algorithm [40] using only hits that were within 10% of the highest matching score. In summary, of the maximum 25 matches any read could have, only matches whose score was within 10% of the highest score were used for taxonomic placement. Taxonomic tables are then computed by counting the number of reads assigned to each node in the taxa tree. Reads matching more than one taxon are placed further up the tree, in the first taxon that is common to all taxa to which the read matched. With this method, ambiguous reads only affect the counts at higher nodes in the taxonomic tree.

The taxa counts were then normalized to adjust for sampling depth by first dividing the abundances in each sample by the number of reads mapping to the plant chromosome in that sample. Then, all values in all samples were multiplied by the mean number of chromosomal plant counts across all samples. This can also be represented by the following formula:

$$X{\mathrm{norm}}_i = {\hat {p}} \cdot \frac{{X{\mathrm{raw}}_i}}{{p_i}}$$

For sample *i* where *X*norm stands for the normalized vector of counts in the sample, ${\bar{\widehat {p}}}$ is the mean number of chromosomal plant counts across all samples, *X*raw_*i*_ is the raw microbial count vector in a sample, and *P*_*i*_ is the number of plant chromosomal reads in that sample.

### Taxonomic correlations and network computation

After computing intermicrobial linear correlations using Pearson’s product–moment and filtering out weakly associated taxa pairs with absolute *r*^2^ value < 0.2 and *p* value < 0.05, network representation was computed with the networkx Python package using the kamada kawai layout function with standard parameters based on the correlation values.

### Plant genotyping

Individual host genotypes were determined using plant associated reads from each metagenome. Reads aligned to the TAIR10 reference genome [36] were filtered to a minimum mapping quality of 20, resulting in an average genome coverage from 15x to 40×. Single nucleotide polymorphisms were called using FreeBayes [41], and resulting VCF files were filtered using custom scripts. SNPs with a minimum alternative count above 3, minimum read depth of 6, and no more than 5% missing data across all samples were kept for downstream analysis.

For determining genotype groups, a genetic distance matrix was computed with ngsDist [42] from the alternative allele count matrix of all SNPs that passed filtering thresholds. This distance matrix was used as input in tsne [43] to visualize sample clustering.

### Adjusting 16S rDNA amplicon data by bacterial load

To adjust the 16S rDNA amplicon dataset to correct for bacterial load, the abundance of each OTU from a sample in the total sum-scaled OTU table can be multiplied by a load scaling factor calculated from the metagenome data for that sample. The simplest load scaling factor is the ratio of all bacteria to plant chromosomal reads in the metagenome sample. If read depth in the metagenome allows, a more precise scaling factor can be calculated based on bacterial families detectable by both methods. Both methods yield similar results in our dataset, because the majority of sequencing reads in both methods fall into bacterial families shared by both methods. We scaled based on bacterial families shared by both methods.

To correct the 16S rDNA dataset by shared taxa in the scaled metagenome dataset, the 16S rDNA dataset was first normalized to 100% in each sample by total sum scaling. The common bacterial families that could be identified by at least a single read in both the metagenomic and 16S rDNA datasets were then identified for each sample, and the sum of read counts in falling in these common taxa was calculated for each sample in both the metagenome and 16S rDNA datasets. The sum of common taxa for each sample in the plant-chromosome-scaled metagenome dataset was divided the sum of reads in these common taxa in the corresponding 16S rDNA table to yield a load scaling ratio. The load scaling factor was multiplied by all the 16S rDNA counts in that sample to produce load-corrected 16S rDNA abundances, closely matching the values obtained from the metagenome. For each sample *i*,

$$\frac{{{\sum} {Mc_i} }}{{{\sum} {Ac_i} }} \times A_i = {\mathrm{load}\,\mathrm{corrected}\,\mathrm{OTUs}}$$

Where ${\sum} {Mc_i}$ is the sum of metagenome reads in common taxa from the plant-chromosome-scaled metagenome table, ${\sum} {Ac_i}$ is the sum of 16S rDNA reads falling in common taxa, and *A*_*i*_ is the full set of 16S rDNA read counts for that sample.

### Comparison of 16S rDNA reads from the metagenome to 16S rDNA V4 amplicons

Metagenome reads from each sample in the batch 3 dataset were mapped to the RDP 16S rDNA training set using phyloFlash [44]. Each metagenome sample yielded an average of 280 mapped 16S rDNA reads, with many yielding fewer than 100 reads. Because for most samples there were too few reads to compare directly with their 16S rDNA amplicon counterparts, samples were pooled to make one aggregate metagenome dataset containing 52,589 16S rDNA phyloFlash sequences. This was then compared with a corresponding 16S rDNA amplicon dataset comprising 52,589 sequences subsampled from the full 16S rDNA dataset. Each sample contributed a matching number of phyloFlash 16S rDNA or amplicon 16S rDNA reads to either the phylFlash 16S rDNA pool or the amplicon 16S rDNA pool, respectively. The family-level relative abundances for these pools were then plotted against each other.

## Results

### Sequencing and analysis approach

To obtain an unbiased picture of microbial diversity and microbial load in wild *A. thaliana* plants, we took a metagenomic shotgun sequencing approach to analyze entire wild phyllospheres. Because plant genomic DNA was expected to dominate such samples, we initially attempted to develop a protocol to enrich the microbial component. To this end, we used genomic DNA from sterilely grown *A. thaliana* plants in excess as bait to remove plant DNA from prepared shotgun libraries. We were unable to find conditions that allowed for consistent, substantial enrichment of microbial sequences (see Supplementary Discussion 1: subtractive hybridization).

We therefore proceeded to shotgun sequence the aerial portions of 275 nonflowering *A. thaliana* individuals from well-characterized locations in southwest Germany [23]. This compilation spanned two different growing seasons (2014/2015 and 2015/2016) with samplings in winter and early spring. Our strategy captured distinct ecological conditions as well as nonoverlapping populations across time due to the winter-annual lifestyle of *A. thaliana* in the region (see “Methods”). Samples were washed three times vigorously with sterile water to remove loosely adhering dust and dirt particles prior to freezing samples on the same day as the harvest at −80 °C. At a later date, total genomic DNA was extracted with a harsh bead-beating procedure to target complete lysis across all domains of life (see “Methods”), and converted into barcoded Illumina short-read sequencing libraries. Plants from the five collections were processed in three batches.

To determine host DNA content, quality-filtered sequencing reads were mapped to the *A. thaliana* Col-0 TAIR10 reference genome [36] with bwa-mem using standard parameters [45]. Sequences that did not map to the reference genome were then translated in silico in all six reading frames and aligned against NCBI’s nonredundant protein database using DIAMOND [38] with standard parameters, and alignments were processed with MEGAN [39, 40].

We reasoned that the number of reads from the plant nuclear genome was highly correlated with diploid cell equivalents and thus fresh weight [46, 47]. We therefore scaled the non-plant read counts to the number of reads that could be mapped to any of the five *A. thaliana* chromosomes. This use of plant chromosomal DNA is analogous to studies that use internal “spike-in” controls calibrated to sample weight or volume [48–50]; our “spike-in” is inherent to our samples (Supplementary Fig. 1). Microbial load calculated in this way correlates well with both qPCR and CFU counting [51].

About half of non-plant reads in each sample could be assigned to microbial taxa. That the number of non-classifiable reads positively correlated with the number of microbial reads suggested that these unclassified reads were mostly from portions of microbial genomes not present in the NCBI nr database, rather than being plant sequences not found in the *A. thaliana* reference genome (Supplementary Fig. 2). To gauge how likely the unclassified reads were to contain sequences missing from the *A. thaliana* reference genome, we mapped these to additional *A. thaliana* genomes assembled from long-read data [52] including five genomes available in house. The number of unclassified reads that mapped to additional plant genomes was unrelated to the quantity of unclassified reads in the sample; even in samples with up to 21% unclassified reads, the fraction of reads that mapped to the additional reference genomes was <1% of the total classifiable plant reads (Supplementary Fig. 2). In other words, across all samples, only a small but consistent percentage of unclassified reads was likely to come from the plant. The rest most likely reflects additional microbial sequences. These sequences may belong to noncoding regions or genes from known taxa that have not been assembled and incorporated into the database. Currently, we cannot easily know how many of the nonclassified, but putative microbial reads reflect highly variable sequences of accessory genomes from known taxa, nor how many reflect the presence of microbial taxa that have not yet had their genomes sequenced. Overall, our results were reminiscent of efforts to classify metagenomic reads from soil and human gut, where more than 50% of reads could not be annotated against known databases [53–55].

To further investigate the possibilities for species-level identification of microorganisms and to search for microbial functions, we attempted metagenome assembly of all samples. We assembled short reads with MEGAHIT [56] (meta-sensitive preset parameter), filtered out contigs shorter than 200 bp, and assessed standard assembly metrics such as N50, N90, mean contig length, and total assembly size (Supplementary Fig. 3). In addition, we mapped reads back to their corresponding assemblies to determine what fraction of each library was effectively being incorporated into contigs. The N50 of contigs that was at least 200 bp long ranged from 500 to 800 bp, with the maximum length of individual contigs in the different assemblies ranging from 6 to 12 kb and the sum of their lengths ranging from 4 to 160 Mb. The mapping rate of short reads to their respective assemblies was only around 25%, representing relatively low average coverage, mostly in the single digits. This is similar to a more successful marine metagenome assembly effort which achieved 30% recruitment into contigs with a similarly low N50 between 300 and 500 bp [57]. We made a further attempt at assembling reads into contigs using metaSPAdes [58]. We tried assembling individual samples separately as well as pooling them by sampling location to increase coverage. This yielded only modest improvements (Supplementary Fig. 4). This difficulty to assemble long contigs was an apparent consequence of high sample diversity (Supplementary Fig. 5). It paralleled the limited success in assembling metagenomes of deeply sequenced soil, where even with over 300 Gb of data, 80% of sequences could not be assembled because of low coverage of individual taxa [53].

To evaluate the reproducibility of our approach and to infer potential biases, we prepared independent libraries for 42 samples. We used the same DNA input for 18 samples, but we also split the plant material after it had been ground in liquid nitrogen and performed independent DNA extractions for 24 samples (Supplementary Fig. 6). The comparison of the microbial components of the resulting sequencing libraries revealed that samples from the same plant grouped together in hierarchical clustering and ordination analyses, both for libraries prepared from the same DNA and libraries prepared from different DNA extractions. The inter-sample distances were similar for both types of replicates.

### Overview of microbial taxa in the metagenomes

**Fig. 1:**
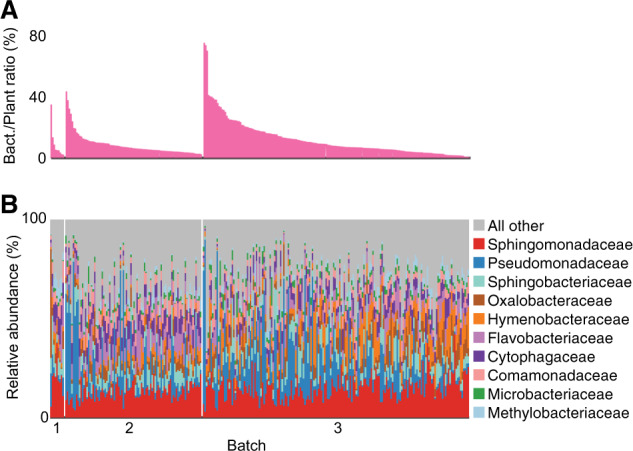
Wild *A. thaliana* leaf microbiomes from shotgun sequencing. **a** Relative abundance of bacterial reads as a fraction of total reads in each sample. **b** Relative abundance of the ten most prevalent bacterial families, computed with plant-chromosome-scaled read counts. Samples are grouped by processing batch in the same order as in **a**.

Overall, we found large variability in the fraction of assignable microbial reads, ranging from 3 to 45% of total read counts in each sample (Supplementary Fig. 7). The vast majority of microbial sequences was identified as bacterial (Supplementary Figs. 2, 8), representing on an average 47 families and an average Shannon Diversity of 25 expected number of families (Supplementary Fig. 9). Taxonomic composition varied across plants (Fig. 1), seasons, and locations (Supplementary Fig. 10). Sphingomonadaceae and Pseudomonadaceae consistently ranked as the most abundant bacterial families (Fig. 1b). Despite both being very common, they behaved very differently across samples: Pseudomonadaceae varied greatly in their relative abundance, with a few samples having substantially higher counts relative to the rest, while the fraction of Sphingomonadaceae reads was much more even across samples (Figs. 1b, 3e and Supplementary Fig. 11).

We had chosen an arbitrary depth of sequencing for our effort, and we therefore wanted to learn how much information would be lost by reducing the number of sequencing reads per sample. We made use of the replicated individuals to this end. We downsampled reads from replicated libraries and compared their taxonomic profiles at the family level. We estimated that ~300,000 non-plant reads constitute a lower bound for robust description of taxonomic profiles (Supplementary Fig. 6). This agrees well with similar estimates for human gut microbiome samples [16], and translates into 7.5 million total reads, or just under 1.12 Gb total sequencing reads per sample for 90% of the dataset.

As a counterpoint to downsampling reads, we were curious how much could be gained by having much deeper sequence coverage from a single plant. Therefore, we processed a single plant that was visibly infected with white rust (*Albugo* spp.) and downy mildew (*Hyaloperonospora arabidopsidis*), and that we in addition left unwashed to further increase the fraction of microbial reads. We sequenced this plant to high depth (~20 Gb), which was 5–20-fold more coverage than the other samples. Fewer than 40% of reads from this sample mapped to the *A. thaliana* reference genome. Similar to our other samples, about half of the remaining reads could be assigned to microbial taxa, with over 90% coming from bacteria (Supplementary Fig. 12). In addition to many *Albugo* spp. and *H. arabidopsidis* reads, we found many of the bacterial taxa already detected in the other samples, and in similar proportions.

### Influence of site, season, and host genetics

A common way to compare composition of microbiomes is based on the Bray–Curtis dissimilarity measure. However, a true distance metric is better suited than a dissimilarity measure for other downstream analyses such as principal component analysis [59]. For distance/dissimilarity measures weighted by taxa abundance, highly abundant taxa can strongly skew results, while low abundance taxa contribute relatively little information.

**Fig. 2:**
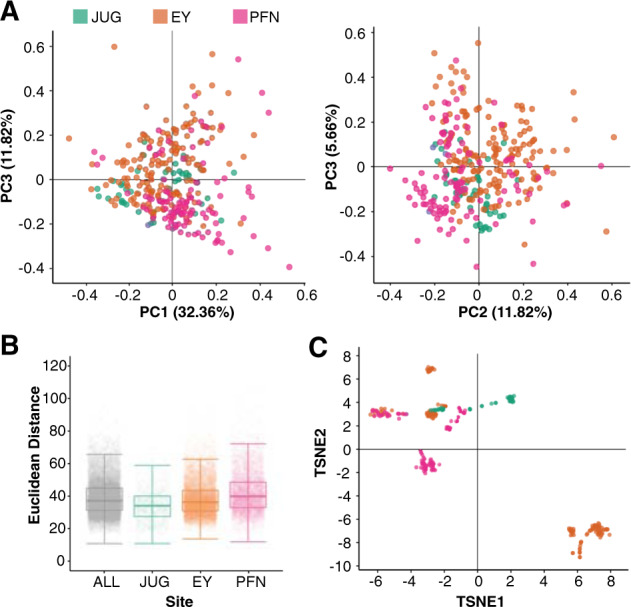
Impact of dominant taxa on microbial community structure. **a** Principal component analysis of the fourth root-transformed count matrix colored by sampling location showing PC1 and PC2, left, and PC2 and PC3, right. **b** Bacterial Euclidean distance distribution across all samples in the dataset (ALL), and from each sampling location (JUG Kirchentellinsfurt, EY Eyach, PFN Pfrondorf). **c** t-SNE map of genetic distances (see “Methods”) with samples colored by location. Distinct genotypes can be identified as clusters of samples; note clear correlation between genetic similarity and sampling site.

To evaluate how the microbiomes of our samples relate to each other, we used, instead of Bray–Curtis dissimilarity, pairwise Euclidean distances and PCA. We first transformed the data by taking the fourth root of the family-level abundance table, including all bacterial, fungal, and oomycete taxa. This transformation corrects for positive skewness in count distribution common in ecological datasets [60], and also decreases the influence of high abundance microbes. No single metadata variable could clearly explain the distributions along the main axes in PCA, although collection site seemed to do best (Fig. 2a, b and Supplementary Fig. 13). Clustering of samples was most clearly driven by the most abundant taxa in each sample, a feature that correlated with collection site. Separation by Pseudomonadaceae or Sphingomonadaceae was apparent when comparing PC1 with PC2, whereas separation by less abundant taxa could be seen when comparing PC2 with PC3 (Supplementary Fig. 14).

Finally, we used the abundant plant reads for host genotyping, using FreeBayes [41] to call close to 1 million SNPs from reads with high-quality mapping to the TAIR10 reference genome. There were several clear clusters of plant genotypes (Fig. 2c) that correlated with sampling site, in agreement with stands of *A. thaliana* in southwest Germany normally hosting only a limited number of genotypes [23]. It is well known that host genotype can influence the composition of the leaf microbiome [61, 62], but as genotype is strongly linked to site in wild populations, both variables are confounded and additional data would be required in order to separate the effects of each.

In an orthogonal analysis, we first classified reads broadly as bacteria, fungi, plants, or unclassified, and compared overall sequence similarity between samples in each class using MASH [63], which measures similarities in k-mer abundance. MASH does not consider the taxonomy of sequences, and therefore provides a sanity check in the sense that the patterns in the data are not dependent on SNP calling, classification, and binning. This classification-independent analysis captured many of the same patterns in the data, with PCoA on MASH distances between bacterial, fungal, plant, or unclassified reads also leading to some degree of clustering of samples by collection site (Supplementary Fig. 15).

### Intermicrobial correlation networks

**Fig. 3:**
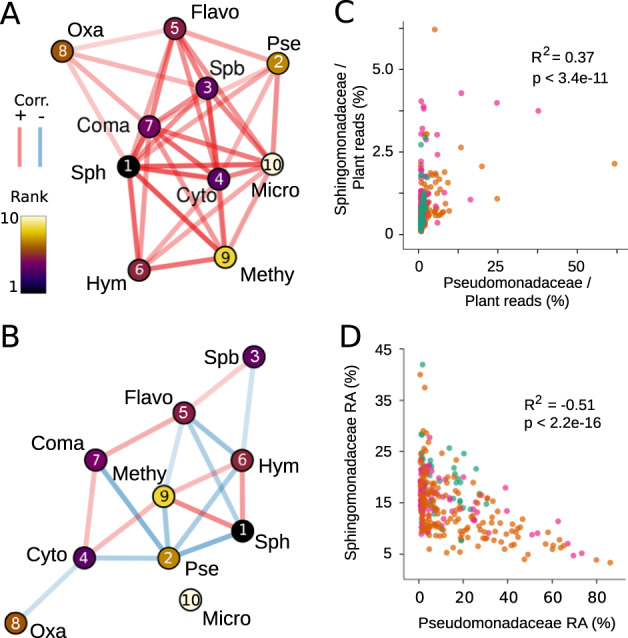
Opposite intertaxa correlations inferred from absolute and relative abundance data. **a**, **b** Correlation networks of the ten most abundant taxa at family level. Nodes represent individual taxa and edges correspond to statistically significant (*p* < 0.05, *R*^2^ > 0.2) Pearson product–moment correlation between taxa across all samples. Colors indicate direction of correlation (red—positive, blue—negative), transparency reflects correlation strength. Labels on top of nodes indicate bacterial families as shown in Fig. 2. Pse Pseudomonadaceae, Sph Sphingomonadaceae, Flavo Flavobacteriaceae, Hymn Hymenobactereaceae, Methy Methlobacteriaceae, Coma Comamonadaceae, Cyto Cytophagaceae, Oxa Oxalobacteraceae, Micro Micrococcaceae, Spb Sphingobacteriaceae. Nodes are colored relative to the mean rank in the dataset (scale left, rounded rank in node). **a** Network based on scaled load data. **b** Network based on relative abundance data. **c** Correlation between plant-scaled Sphingomonadaceae and Pseudomonadaceae bacterial load. **d** Correlation between Sphingomonadaceae and Pseudomonadaceae relative abundance.

Shotgun sequencing provides a minimally biased estimation of the true abundance of microbes in a microbiome sample. We examined microbial abundances across all samples and under different data transformations to understand colonization patterns and potential intermicrobial relationships. We first made a map of pairwise linear correlations between all bacterial families that passed filtering thresholds (1000 assigned reads per family in at least ten samples) using plant-scaled data (equal plant chromosomal reads), relative abundance data, and fourth root of plant-scaled data. Only co-occurrences with a Pearson correlation coefficient >|±0.2| and with a *p* value lower than 0.05 after Student’s *t* test were used (Supplementary Fig. 16). On average, a given taxon was positively correlated with 13 other taxa, but this was heavily skewed toward microbes present in many samples, as correlations between taxa only seen in a handful of plants were usually not significant. In addition, bacterial load varied widely across samples, and it was positively associated with the abundance of sequences associated with each taxon (Supplementary Fig. 17). This resulted in only positive correlations between taxa (Fig. 3a). That is, a high bacterial load meant a higher abundance of nearly all taxa, although some taxa such as Pseudomonadaceae contributed more to microbial load than others. When applying fourth root transformation to the plant-scaled microbial counts, the same trend as in untransformed data was observed for all taxa, the only difference being increased correlation values and an increased number of correlated taxa pairs.

If bacterial data in all samples are transformed to relative abundance prior to analysis—a necessity for data without internal standards such as most amplicon data—the table becomes compositional, and taxa abundance estimations become constrained because the sum of all taxa is constant. This greatly confounds the directionality of correlation. For example, when one taxon becomes dominant, it will automatically reduce the abundances of all other taxa, which easily misleads to the conclusion that the population of the dominant taxon grows at the expense of the others. When a relative abundance transformation was applied to our dataset, many of the positive correlations between taxa either disappeared or became negative, including the one between Pseudomonadaceae and Sphingomonadaceae (Fig. 3). This trend was observed across the entire dataset as well as in individual sampling sites (Supplementary Fig. 18). Using only a table of relative abundance, it would be tempting to see this correlation as evidence for widespread antagonism between these families in the wild. Indeed, antagonism between *Pseudomonas* and *Sphingomonas* is known to occur in laboratory conditions [64].

Recognizing this pitfall, there are several mathematical transformations that have been developed to help avoid these spurious conclusions [11–13, 65, 66]; for example, the centered log ratio (CLR) relates the abundance of each taxon in a sample to the geometric mean of all taxa in that sample. In addition to removing the range constraint of relative abundance in this manner, by further taking the logarithm, the CLR greatly reduces the ability of highly abundant outliers to distort the values of other taxa. The CLR correctly identifies a positive correlation between Pseudomonadaceae and Sphingomonadaceae in our dataset, although it overestimates the strength of the correlation (*R*^2^ = 0.30 vs. *R*^2^ = 0.07).

### Concordance between metagenome and amplicon data

**Fig. 4:**
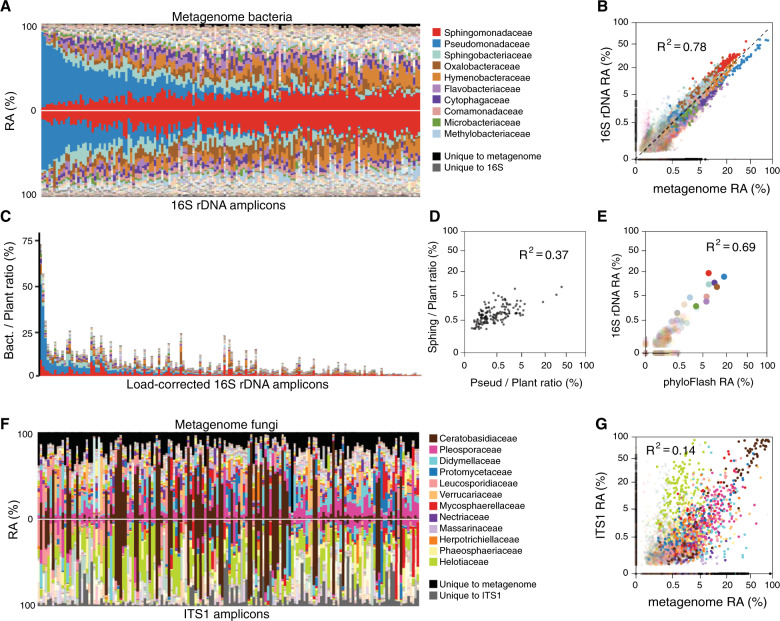
Enhancing utility of metagenome data with parallel amplicon data. **a** The relative abundances (RA) of bacterial families as determined by the shotgun metagenome pipeline (top) mirrored against bacterial families as determined by the 16S V4 rDNA amplicon pipeline (bottom) for batch 3 plants (columns). Samples in all panels are ordered by the abundance of Pseudomonadaceae (blue) in the metagenome. Taxa unique to the metagenome are shown in black, those unique to amplicons in dark gray. **b** The two datasets from **a**, fourth root-transformed and shown as a scatterplot. The dotted line represents 1:1 correlation. **c** The amplicon data from **a**, bottom, scaled by common taxa shared between the metagenome and amplicon data. **d** The data in **c**, fourth root transformed and shown as a scatterplot. **e** Scatterplot of fourth root-transformed bacterial family abundances, comparing 16S rDNA amplicon data with 16S rDNA sequences detectable in the metagenome (using phyloFlash). Same color scheme for families as in **a**–**d**. **f** The RA of fungal families as determined by the shotgun metagenome pipeline (top) mirrored against fungal families as determined by the ITS1 rDNA amplicon pipeline (bottom) for batch 3 plants (columns). Samples are ordered as in **a**. Taxa unique to the metagenome are shown in black, those unique to amplicons in dark gray. **g** Scatterplot of fourth root-transformed data from **f**.

In order to contrast information from metagenome and amplicon sequencing, we focused on the largest batch, batch 3, with 176 samples. We PCR amplified and sequenced the V4 region of bacterial 16S rDNA and the fungal ITS1 region for these samples. Because the V4 16S rDNA sequence of *A. thaliana*-associated cyanobacteria is indistinguishable from that of chloroplasts, reads with cyanobacteria assignments were ignored and cyanobacteria reads were also removed from the metagenome dataset. The agreement between assignment of bacterial families based on 16S rDNA amplicons and metagenomes was very high (Fig. 4a), with an overall Pearson coefficient of correlation *R*^2^ of 0.78 on fourth root-transformed data (Fig. 4b). Among the top taxa, compared with metagenomics estimates, 16S rDNA estimates were slightly lower for Pseudomonadaceae, and slightly higher for Sphingomonadaceae, Sphingobacteriaceae, and Oxalobacteraceae (Fig. 4a, b). In a complementary comparison, we extracted only the 16S rDNA sequences from the metagenome reads and classified them using phyloFlash [44]. When plotted against 16S rDNA amplicons that had been subsampled to match the metagenome 16S rDNA read counts, the overall correlation and overestimation/underestimation trends were the same as for the comparison of amplicons with all metagenome reads (Fig. 4e, compare with Fig. 4b).

The concordance between fungal families deduced either from ITS1 amplicons or metagenomes was weaker than for bacterial families (Fig. 4f, g), with a Pearson coefficient of correlation *R*^2^ of 0.14. Several factors could potentially explain this difference. First, because fungi are less abundant overall, their quantification is based on fewer sequences and therefore noisier. In agreement, the Pearson correlation coefficient *R*^2^ of metagenome versus amplicon data for the most abundant fungal family, Ceratobasidiaceae, was much higher than the average for fungi, with *R*^2^ = 0.88. Among other families, the Helotiaceae were especially poorly correlated, and were far more abundant in the ITS1 data (Fig. 4f, g). While this could be due to a bias of the ITS1 primers for this family at the exclusion of others, it could also be that metagenome sequences from this family are more often erroneously assigned to other, sequence-related families, deflating counts of Helotiaceae. Another potential source of discrepancy between the datasets could come from fungal genomes varying more widely than bacteria in size, and rDNA copies being only poorly correlated with fungal genome size [67]. This can introduce biases because species with larger genomes would appear to have higher abundances in metagenomes due to more mapped reads. On the other hand, because larger genomes may not have more rDNA copies, ITS1 amplicon counts are less affected by differences in genome size. Additionally, because a much smaller fraction of fungal genomes—as compared with bacterial genomes—codes for proteins, and because gene number in fungi varies much less than genome size, many fungal sequences in the metagenome may not be represented in the protein databases used for classification and quantification of reads.

The close concordance between metagenome and 16S rDNA relative abundances enables the scaling of 16S rDNA amplicon data based on bacterial load obtained from metagenome data. Using amplicons, abundant plant host DNA is much more easily blocked using either PNA oligomers [27] or special blocking primers [31], meaning that for samples with high plant DNA content, amplicon sequencing can sample many more microbes at a lower cost than metagenome sequencing. Entries in amplicon databases currently represent the taxonomic breadth of microbes more evenly than whole genome or protein databases, and therefore may provide more consistent classification [68]. In addition, we estimate that relatively shallow metagenome sequencing of 30,000 reads, much <300,000 needed to assign microbial taxonomy, is sufficient to find enough classifiable reads with which to estimate the bacterial load of a sample (Supplementary Fig. 19). Low-cost shotgun library preparation methods [69] in particular make a hybrid approach, in which amplicon sequencing is combined with low-depth shotgun sequencing, attractive. We used the bacterial load as calculated from plant-scaled metagenome data to adjust the abundances of 16S rDNA data to reflect estimated loads (Fig. 3c). As would be expected from the close correlation of 16S rDNA and metagenome data, the adjusted 16S rDNA data accurately captured the slightly positive correlation between Pseudomonadaceae and Sphingomonadaceae across the dataset (Fig. 3d).

## Discussion

Describing the phyllosphere-associated microbial community in the context of natural or field cultivated plant populations is of fundamental importance for understanding and designing microbial interventions in conservation and agriculture. For years, as in studies of other microbial communities, studies of the phyllosphere microbiota have been approached qualitatively via isolation and culture of specific leaf microbes [70–72]. With the advent of high-throughput amplicon and short-read sequencing, it has become easier to address the larger community of taxa that interacts with its host in a quantitative manner. Here, we investigated the advantages as well as the limits of whole-metagenome shotgun sequencing to study the leaf microbiome.

A first finding was that de novo assembly produced few longer sequences (Supplementary Figs. 3–5), and thus did not provide advantages over directly mapping short reads to reference databases for taxonomic assignment. Using taxonomic assignments inferred from short reads directly, we found that the relative abundance of bacterial taxa among all bacteria was highly consistent with 16S rDNA amplicon measurements, with the highest correlation seen for the most abundant taxa (Fig. 4). The relative abundance of fungi in the metagenome correlated less well with the ITS1 amplicons, which could be explained at least in part by their lower abundance as well as more complex genomes of fungi compared with bacteria. Nevertheless, the metagenome data clearly showed that fungi are ubiquitous in *A. thaliana* leaves, even though they are usually only a minor part of the overall *A. thaliana* phyllosphere microbiome.

We used shotgun sequencing to estimate microbial load across samples, which varied substantially. The absolute estimates allowed us to reveal the extent to which normalizing bacteria or fungi to a common value via rarefaction or by total sum scaling, as is common practice, may mislead researchers to equate an increase or decrease in relative abundances with a change in absolute abundances. Indeed, without microbial load information, some phenotypes may not be detectable [73].

We used load-corrected bacterial taxonomic profiles to explore similarities and differences among the microbiomes. We did not detect a strong individual influence of either environment or host genetics on the structure of leaf communities, although some combination of both contributed (Fig. 2, Supplementary Fig. 13). We found that the most abundant taxa in a sample predicted the community structure of the other microbes in the sample (Supplementary Fig. 14. In our natural host populations, there was not enough genetic diversity at each site to allow us to disentangle the effects of site and host genotype on microbiome composition. Whether variation between genetically identical hosts reflects only stochastic effects, or variation in microenvironment, needs to be determined.

An important goal of microbial ecology is to uncover specific interactions between community members, which can point to key taxa that have a major effect on community composition [74, 75]. Correlation networks present a valuable tool for such investigations; we have demonstrated that correlations based on relative abundances can lead to very different networks than correlations based on absolute abundances (Fig. 3). In our specific case, relative abundance data had suggested that Pseudomonadaceae and Sphingomonadaceae, two of the most common bacterial families typically found in a leaf microbiome, were negatively correlated, when in fact they were positively correlated, as demonstrated with the absolute abundance data. We observed such patterns for several other taxa pairs as well. It is striking that statistically significant correlations were always positive in our dataset when using load-corrected data. This could simply reflect generally stable relationships between community members, such that they tended to succeed or fail together as they colonized the plant.

We also explored the usefulness of compositional data transformations such as CLR. While these are an improvement over naive approaches, we found them to be lacking when compared with the use of true absolute abundance information, and we must conclude that there is no substitute for direct information about microbial load. We note that sometimes habitats can become truly compositional once the carrying capacity of the habitat has been reached, at which point the increase of one taxon can only come at the expense of other taxa. Transformations that mask the effects of compositionality would thus hide the underlying biology. And although it may be possible to estimate the absolute extent of an infection from compositional data, this becomes a complicated problem and remains at best a proxy.

In conclusion, we have demonstrated the advantages of using metagenome shotgun sequencing either alone or in combination with 16S rDNA and ITS1 amplicon sequencing for measuring microbial communities in *A. thaliana* leaves. Modest read depth, as few as 300,000 non-plant reads per sample (Supplementary Fig. 19), is sufficient to enable quantitative taxonomic assignment that is comparable to amplicon sequencing, and 30,000 total reads per sample is sufficient to robustly quantify microbial load. In addition, it turns out once more that the small, ~135 Mb genome of *A. thaliana* is a substantial advantage, as it may currently be cost prohibitive to extend our direct metagenomic approach to other species.

It seems reasonable to assume that microbial load is largely independent of plant genome size. This would mean that in maize, with a 2.5 Gb genome, almost 20× time more sequencing would be required than in *A. thaliana* with its 0.13 Gb genome, even though this is likely an overestimate, because plants with larger genomes tend to have larger cells [76], each of which could potentially support more microbial cells. Nevertheless, while the microbial load on other species remains to be investigated, this is yet another reason to use *A. thaliana* (or other species with relatively small genomes) for microbiome studies in ecological settings. On the other hand, the hybrid approach of using lower coverage metagenome data to estimate microbial load and to use this information to scale amplicon data may cost-effectively support endophytic analysis of plants with larger genomes as well. It is worth noting more generally that differences in host cell size or host cell density could interfere with interpretation of microbial load as calculated by sequencing, so caution should be used when attempting to compare data across diverse hosts. Finally, we do not expect the lack of a high-quality reference genome to be a serious impediment. Using expressed sequence tags or the genome of a related species as mapping reference, many fewer reads will map, but from those that do it should still be possible to scale host reads in each sample and correct the microbial data.

## Supplementary information

supplementary material

## Data Availability

All data in this manuscript has been deposited in the European Nucleotide Archive (ENA). It can be accessed under the project number PRJEB31530 at https://www.ebi.ac.uk/ena.
